# Supplementary material for: Comparative multiomics analysis of cell physiological state after culture in a basket bioreactor
Source: Sci Rep. 2022 Nov 23;12:20161. doi: 10.1038/s41598-022-24687-4 (PMC9686226; doi:10.1038/s41598-022-24687-4)
Supplement: Supplementary file 1 — Supplementary Information 1. [file 41598_2022_24687_MOESM1_ESM.zip › raw data/Transcriptomic raw data/7.AS/2.dasplot/TE1vsTC1/SE/3_LOC119624861_NW_023666039.1_17590734_17590862_-@NW_023666039.1_17528638_17528809_-@NW_023666039.1_17498048_17498175_-.pdf]

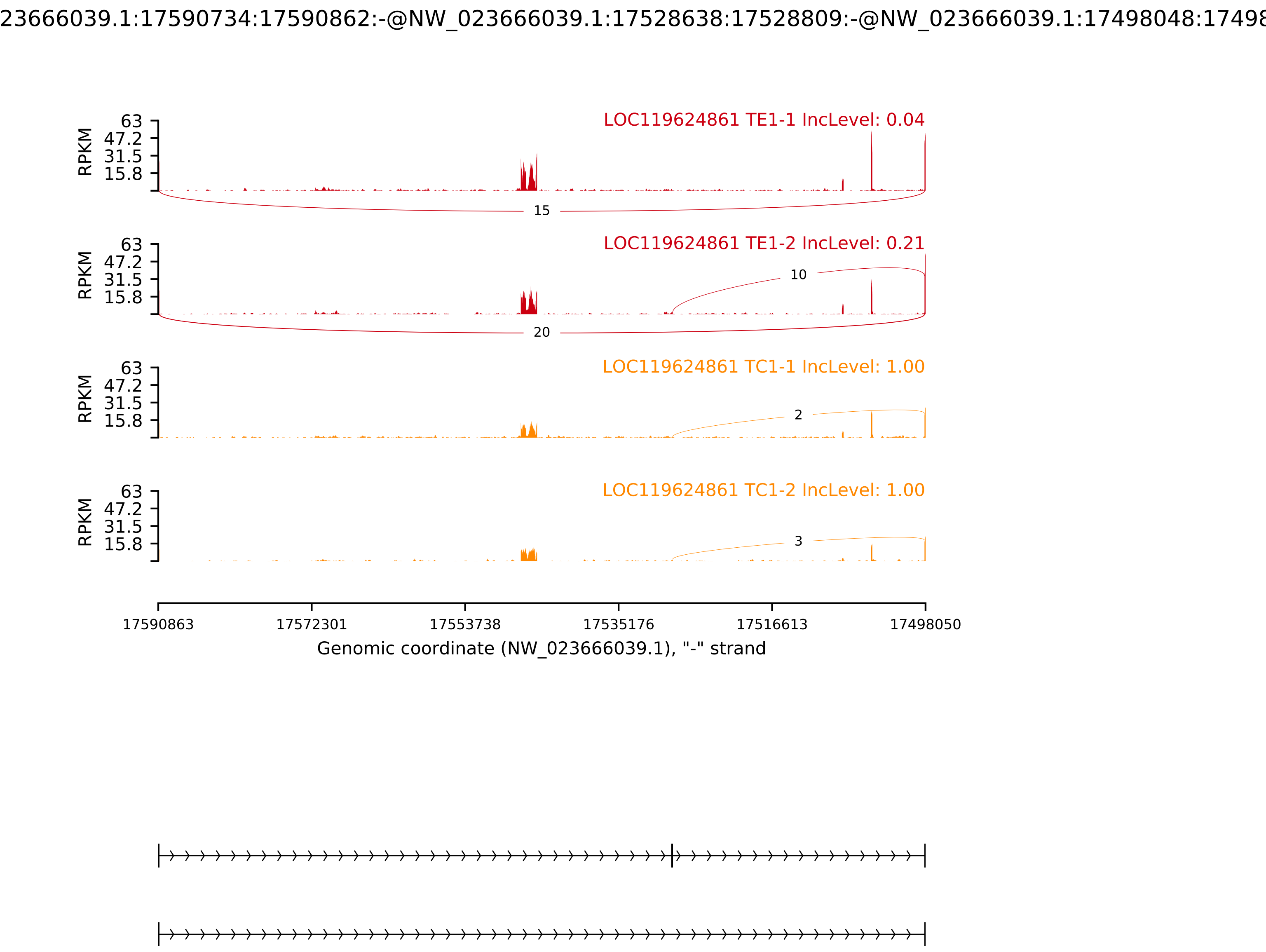

23666039.1:17590734:17590862:-@NW_023666039.1:17528638:17528809:-@NW_023666039.1:17498048:1749
LOC119624861 TE1-1 IncLevel: 0.04
LOC119624861 TE1-2 IncLevel: 0.21
LOC119624861 TC1-1 IncLevel: 1.00
LOC119624861 TC1-2 IncLevel: 1.00
RPKM
63
47.2
31.5
15.8
15
10
20
2
3
17590863
17572301
17553738
17535176
17516613
17498050
Genomic coordinate (NW_023666039.1), "-" strand
